# Case Report: Postmortem brain and heart pathology unveiling the pathogenesis of coexisting acute ischemic stroke and electrocardiographic abnormality

**DOI:** 10.3389/fcvm.2023.1200640

**Published:** 2023-06-14

**Authors:** Yorito Hattori, Shuhei Ikeda, Manabu Matsumoto, Naoki Tagawa, Kinta Hatakeyama, Masafumi Ihara

**Affiliations:** ^1^ Department of Neurology, National Cerebral and Cardiovascular Center, Suita, Osaka, Japan; ^2^ Department of Pathology, National Cerebral and Cardiovascular Center, Suita, Osaka, Japan

**Keywords:** cardiocerebral infarction, cardioembolic stroke, myocardial infarction, atrial fibrillation, coronary artery embolism

## Abstract

Electrocardiography abnormalities have been occasionally reported at the onset of stroke. Simultaneous electrocardiographic abnormalities and stroke require a rapid differentiated diagnosis among several diseases. However, direct causal relationships remain unclear. A 92-year-old woman presented to our emergency department in a sudden-onset coma. The patient suffered from huge acute ischemic stroke with bilateral internal carotid artery occlusion assessed by brain magnetic resonance imaging, and her electrocardiography showed ST-segment elevation at II, III, aVF and V4–6, and atrial fibrillation (AF). However, the etiology of the medical condition was clinically unknown. Eventually, the patient died on day 4 of hospitalization before the diagnosis could be completed. Therefore, an autopsy was performed to investigate pathological findings after obtaining informed consent from the family. A postmortem pathological evaluation demonstrated that fibrin mural thrombi in the left atrial appendage (LAA), and the cerebral and coronary arteries possessed CD31-positive endothelial cells, and CD68-positive and CD168-positive macrophages in a similar fashion, suggesting the fibrin thrombi observed in the three sites implicated to be identical. We concluded that nearly concurrent cerebral and coronary artery embolism because of the fibrin thrombi in LAA developed by AF. Simultaneous cerebral infarction and myocardial infarction are referred to as cardiocerebral infarction (CCI), a rare disorder for which clear pathomechanisms remain unknown, although several mechanisms of CCI have been proposed. We first revealed the clear pathology of CCI using the autopsy. Additional pathological studies are warranted to establish clear pathomechanisms and preventive strategies of CCI.

## 1. Introduction

Bidirectional interactions between the cardiovascular and nervous systems have gotten more attention and are becoming increasingly important (1). Electrocardiography (ECG) abnormalities have been occasionally detected at the onset of stroke. Insular cortical damage such as stroke is linked to QT dispersion (2), negative T-waves (3), and atrial fibrillation (AF) (4, 5). Regarding ST-segment changes among ECG abnormalities, we must consider the following as differential diagnoses: (i) cardiac changes induced by stroke such as takotsubo cardiomyopathy or other cardiac conditions due to insular stroke, and (ii) cardioembolic stroke induced by intraventricular thrombi due to acute myocardial infarction (AMI) or takotsubo cardiomyopathy, and concurrent coronary-cerebral artery embolism induced by intra-atrial thrombi due to AF.

Among the abovementioned differential diagnoses in patients with acute stroke and ST-segment changes, cardiocerebral infarction (CCI) is one of the representative diseases. The simultaneous occurrence of AMI and acute ischemic stroke (AIS) is rare in CCI (6, 7). There are several possible mechanisms of CCI, which can be classified into four categories: (i) conditions causing concurrent cerebral–coronary infarction, (ii) cardiac conditions such as intraventricular thrombi due to wall akinesis or hypokinesis causing cerebral infarction, (iii) cerebral infarction causing AMI due to brain–heart axis dysregulation (8), and (iv) systemic prothrombotic state with hematologic disorders such as polycythemia vera and high plasma factor VIII levels (9). However, the clear pathomechanisms of CCI have not been studied with pathological findings.

Here we successfully revealed a part of causal relationships in a patient with AIS and ST-segment elevation via a postmortem autopsy.

## 2. Case presentation

**Figure 1:**
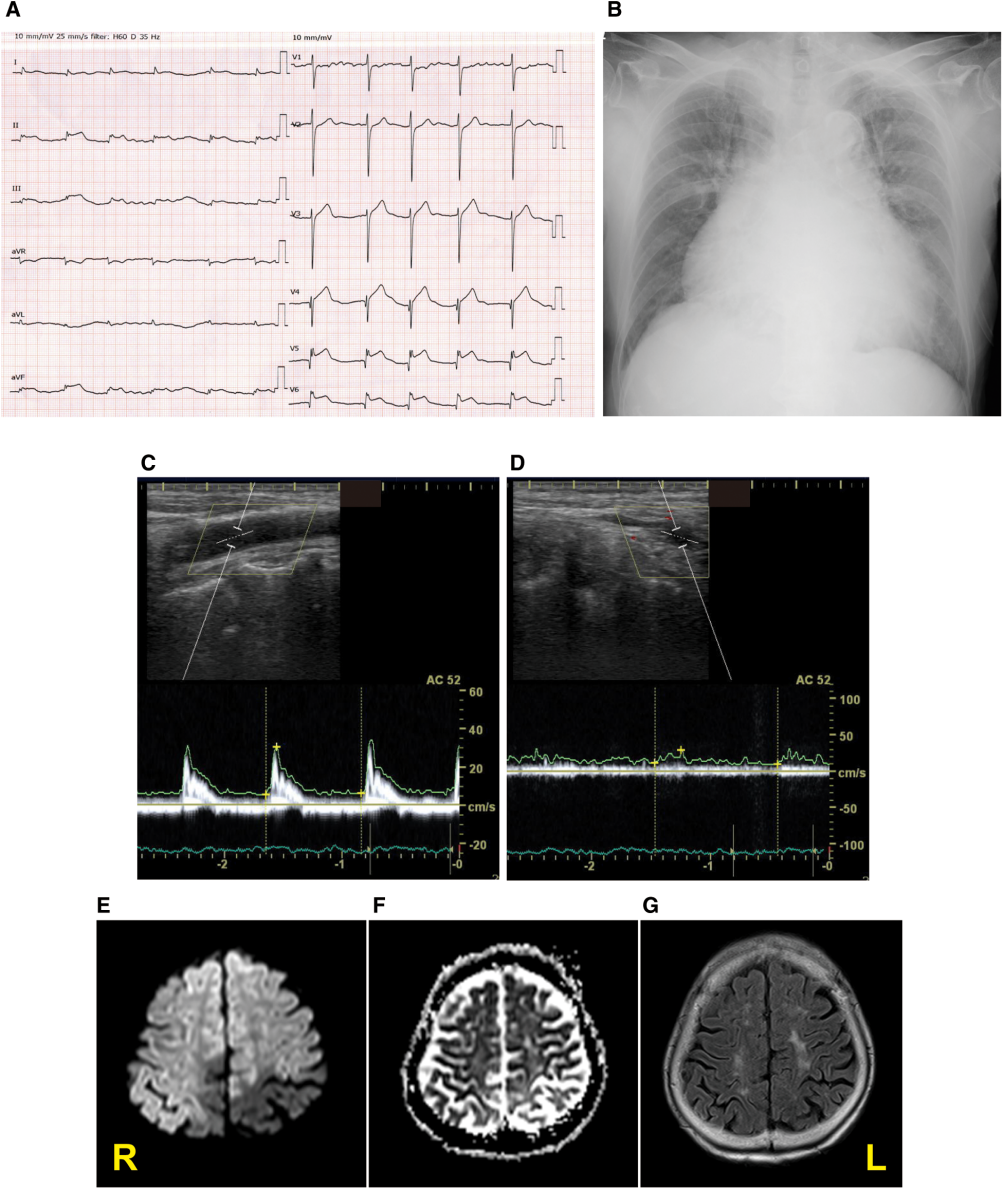
Diagnostic testing performed on admission. Twelve-lead electrocardiography (**A**), chest x-ray (**B**), pulse-wave carotid ultrasonography with Doppler in the left common carotid artery (**C**) and internal carotid artery (ICA) (**D**), and the diffusion-weighted magnetic resonance imaging (MRI) (**E**), apparent diffusion coefficient map (**F**), and fluid-attenuated inversion recovery MRI (**G**) displaying acute bilateral ICA territory infarction. R stands for right; L stands for left.

**Figure 2:**
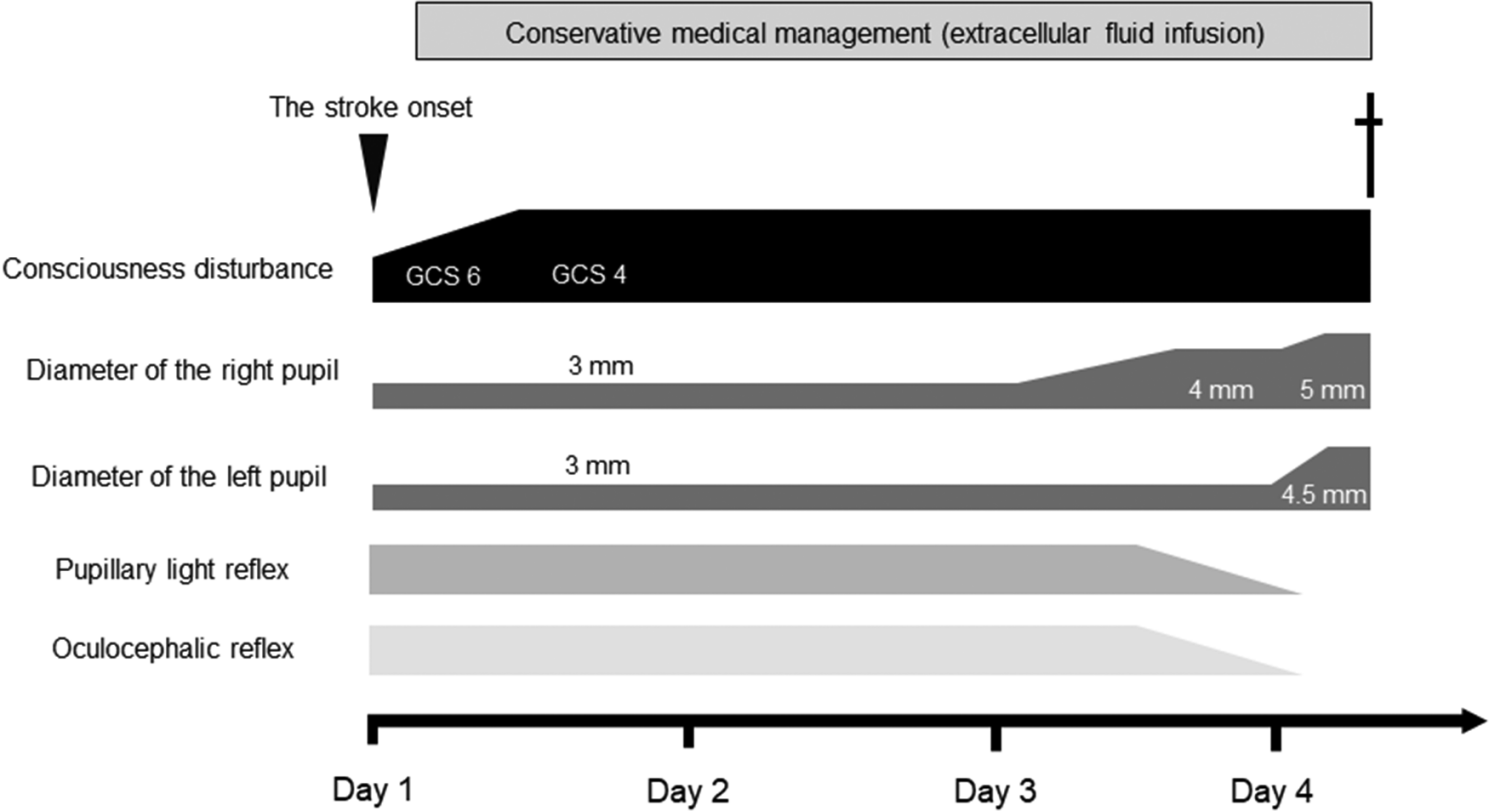
Clinical course of the patient after stroke onset. GCS, Glasgow Coma Scale.

A 92-year-old woman presented to our emergency room with a sudden-onset coma. A family member witnessed that she suddenly fell down on the floor while they were talking. Her eyes were closed, and she had lost consciousness. Although her medical history included chronic AF, hypertension, and aortic valve stenosis, but she did not take any anticoagulants. Her blood pressure was 205/102 mmHg. Neurological examination revealed severe disturbance of consciousness. The patient was mute and could not follow any command. Her Glasgow Coma Scale score was 6 (E1V2M3). The pupil size was normal (3/3 mm), and the oculocephalic reflex showed a positive response; however, roving eye movement was noted. This indicated that the cerebral cortex was impaired while the brainstem was functionally preserved. The pupillary light reflex was prompt bilaterally. Motor assessment revealed flaccid tetraplegia, and the Babinski reflex was bilaterally positive. The National Institutes of Health Stroke Scale score was 34. Blood test revealed 7,600/µl in white blood cell, hemoglobin was 13.4 g/dl, aspirate transaminase was 22 IU/L, alanine aminotransferase was 9 IU/L, sodium was140 mEq/L, glucose was 116 mg/dl, serum creatinine was 0.88 mg/dl, C-reactive protein was 0.04 mg/dL, brain natriuretic peptide was1035.5 pg/mL, and D-dimer was 18.5 µg/mL. A chest x-ray showed cardiomegaly, 72.0% in cardio–thoracic ratio, and ECG revealed AF with ST-segment elevation at II, III, aVF and V4–6 (Figures 1A,B). Echocardiography showed hypokinesis of the anterior and inferior walls. Carotid ultrasonography exhibited a reduction in the end-diastolic velocity (right, 4.9 cm/s; left, 6.3 cm/s) and increased pulsatility index (2.2;2.3) at the bilateral common carotid arteries and did not detect pulse-wave Doppler in the bilateral internal carotid artery (ICA), suggesting that the bilateral ICA was occluded (Figures 1C,D). On brain non-contrast magnetic resonance imaging (MRI), diffusion-weighted MRI (Figure 1E) and apparent diffusion coefficient map (Figure 1F) showed almost the same signal intensity in the infarcted lesions of the bilateral ICA territories, whereas fluid-attenuated inversion recovery revealed no such lesions with a high signal intensity (Figure 1G), demonstrating acute cerebral infarction in the bilateral ICA territories with a nearly simultaneous onset. These clinical findings suggested that acute massive ischemic stroke due to near-simultaneous bilateral ICA occlusion occurred. Following admission, we carefully explained to the family members that reperfusion therapy such as the administration of recombinant tissue-type plasminogen activator, or mechanical thrombectomy was not suitable for the massive cerebral infarction because of the risk of hemorrhagic transformation. Moreover, coronary angiography could not be performed because her neurological prognosis was poor, and the condition would unfortunately be irreversible. Accordingly, conservative medical management was recommended. The family members understood our explanations, and agreed on conservative management after deep deliberation. No reperfusion therapy or coronary angiography was performed. On day 3, her right pupil dilated to 4 mm. On day 4, the patient vomited once. Her pupils further dilated bilaterally to 5.0/4.5 mm, and the oculocephalic and pupillary light reflexes were absent. Her blood pressure and heart rate also gradually decreased. Eventually, the patient died on day 4 of hospitalization before the diagnosis was completed (Figure 2). Clinical differential diagnoses included three categories: (i) conditions leading to concurrent cerebral–coronary infarction in which AF could induce both cardioembolic stroke and coronary artery embolism; (ii) cardiac conditions leading to cerebral infarction, such as intraventricular thrombi due to ST-elevation myocardial infarction or takotsubo cardiomyopathy with ST elevation; and (iii) cerebral infarction leading to AMI through brain–heart axis dysregulation (e.g., insular ischemic stroke). Thus, the etiology of the thrombi and the relationship between AIS and cardiac dysfunction with ST-segment elevation were unclear.

**Figure 3:**
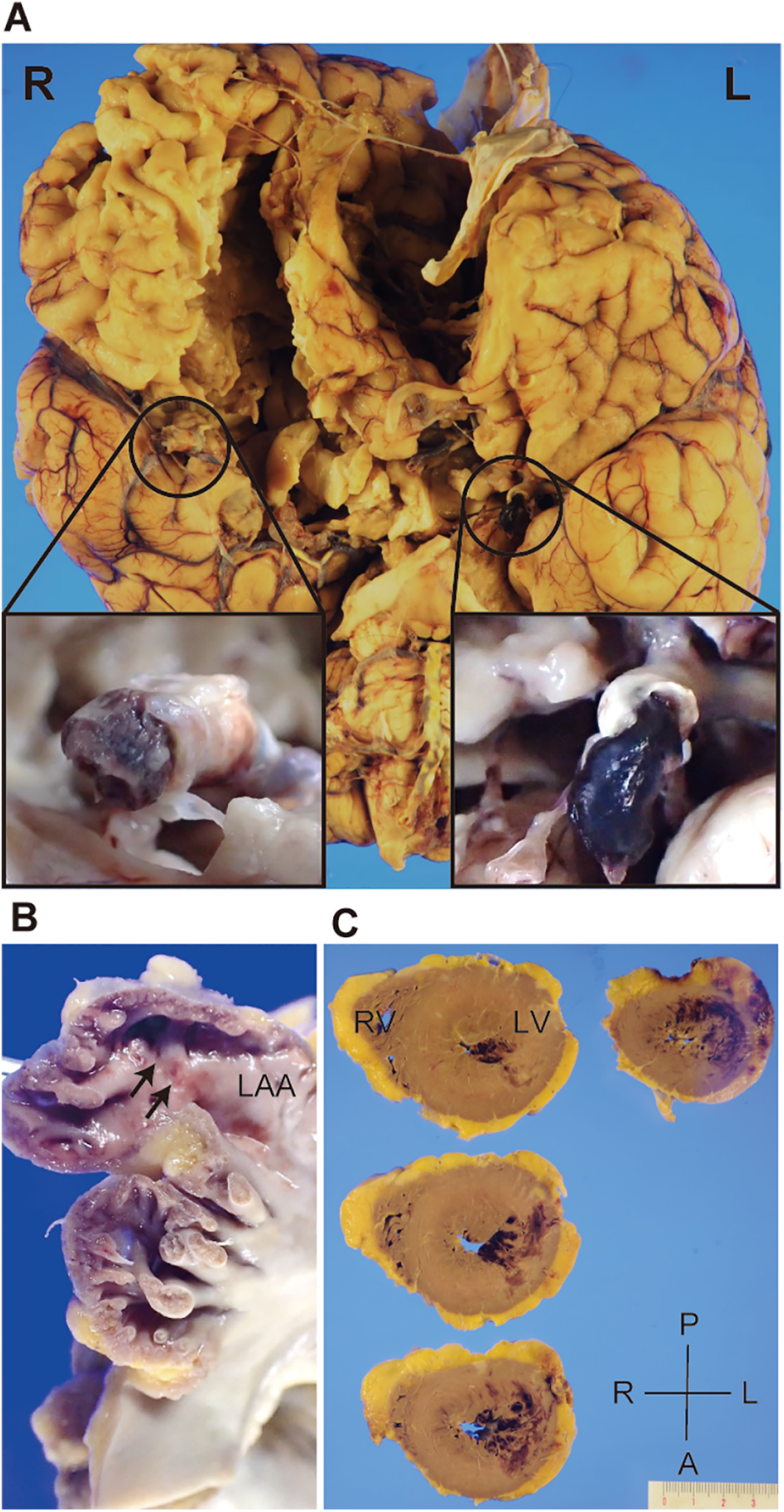
Gross appearance of the patient’s brain and heart during autopsy. Representative pictures depicting bilateral internal carotid artery (ICA) filled with thromboemboli (**A**), mural thrombi in the left atrial appendage (LAA) (**B**, arrows), and acute myocardial hemorrhagic infarcts in the lateral-posterior wall of the left ventricle (**C**). R, right; L, left; A, anterior; P, posterior; RV, right ventricle; LV, left ventricle.

**Figure 4:**
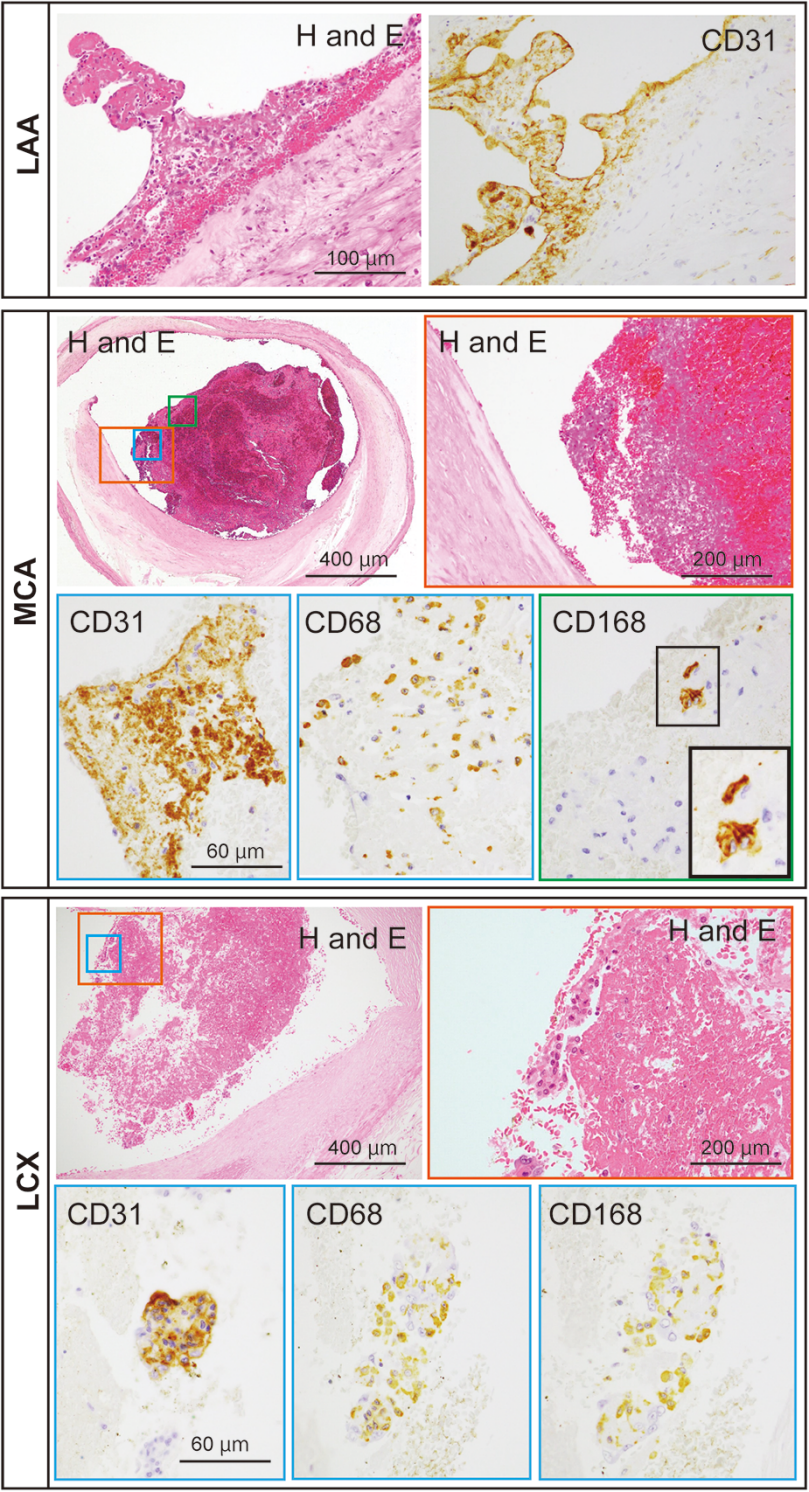
Representative microscopic images of the thromboemboli at the patient’s left atrial appendage (LAA), middle cerebral artery (MCA), and left circumflex artery (LCX). The thromboemboli in the LAA, MCA, and LCX were stained with hematoxylin and eosin (H and E), for CD31, CD68, and CD168. Colored rectangles with lowest magnification in the MCA and LCX are magnified to the pictures surrounded with each colored rectangle. Inset in CD168 of the MCA represents the enlargement of the area outlined by the black rectangle.

An autopsy was performed to explore pathological findings after informed consent was obtained from the family. Macroscopic autopsy examination revealed bilateral ICA occlusion with red thrombi (Figure 3A), mural thrombi in the left atrial appendage (LAA) (Figure 3B), and acute myocardial hemorrhagic infarcts in the lateral-posterior wall (Figure 3C), which suggested the occurrence of coronary artery embolism and recanalization. Microscopic examination revealed thrombi with abundant cell-lytic changes on the wall of the LAA, in the middle cerebral artery (MCA), and in the left circumflex artery (LCX) (Figure 4). In these three sites, the thrombi included CD31-positive cells, and those in the MCA and LCX included CD68- and CD163-positive cells similarly (Figure 4). The autopsy results suggested that the fibrin thrombi observed in the three sites were identical. We eventually concluded CCI where the fibrin thrombi in the LAA developed by AF nearly simultaneously migrated into the bilateral ICA and coronary arteries, leading to near-concurrent cardioembolic stroke and coronary artery embolism.

## 3. Discussion

The patient was conclusively diagnosed as near-concurrent cardioembolic stroke and coronary artery embolism because of AF. A total of 5,953 patients with AIS had been admitted to our hospital between January 2011 and March 2020. Of them, six patients were identified with CCI (0.1%, 71–94 years old, five women) (Table 1). Five patients had cardioembolic stroke. All five patients diagnosed as cardioembolic stroke had AF, but no patients had received oral anticoagulation before the events. Some associations between AMI and/or AF, and AIS were implicated, however, precise associations regarding CCI were not evident. Shibata et al. proposed diagnostic criteria for a clinical diagnosis of coronary artery embolism attributing to AMI (10). According to the criterion, only Patient 1 was equivalent to definite coronary artery embolism, whereas most of the patients, including Patient 6 (the current case) were dissociated with the clinical diagnoses as CCI with coronary artery embolism (Table 1). Thus, the criterion was still inappropriate to precise diagnosis as CCI subtype of concurrent cerebral and coronary artery embolism, suggesting that pathological findings are of importance for precise diagnosis.

**Table 1:** Clinical data on patients suffering from a cardiocerebral infarction.

Patient	1	2	3	4	5	6
Age	73	71	84	68	94	92
Sex	F	M	F	F	F	F
Premorbid ATA	None	ASA	None	ASA, CLP	None	CLS
Clinical diagnosis of CI subtype	CES	LAA	CES	CES	CES	CES
Clinical diagnosis of AMI subtype	CE	Atherosclerosis	CE	CE	CE	CE
AF	+	−	+	+	+	+
ECG findings	STEMI	non-STEMI	STEMI	non-STEMI	non-STEMI	STEMI
Treatment for CI	rt-PA, MT	ASA	Conservative treatment	rt-PA, MT	rt-PA, MT	Conservative treatment
Treatment for MI	Heparin	Heparin	Conservative treatment	ASA	rt-PA	Conservative treatment
mRS at discharge	2	4	5	4	3	6
Definite CE (10)	+	−	−	−	−	−

AF, atrial fibrillation; ATA, anti-thrombotic therapy; CI, cerebral infarction; AMI, acute myocardial infarction; ECG, electrocardiography; mRS, modified Rankin scale; CES, cardioembolic stroke; CE, coronary artery embolism; ASA, acetylsalicylic acid; LAA, large-artery atherosclerosis; CLP, clopidogrel; rt-PA, recombinant tissue plasminogen activator; MT, mechanical thrombectomy; STEMI, ST-segment elevation myocardial infarction; CLS, cilostazol.

In the autopsied case, the thrombi with CD31-positive endothelial cells adhered to the LAA wall and were also found inside the lumens of the MCA and LCX, implying that the thrombi with endothelial cells of the LAA wall occluded both the MCA and LCX. Furthermore, in the MCA and LCX, CD68-, and CD163-positive macrophages were found inside the thrombi, indicating that the thrombi formed at the similar time (11). Thus, it was clear that the current case had a near-concurrent cerebral–coronary artery embolism. How fibrin thrombus age is determined has been assessed in animal and human studies. Macrophages rises in experimental fibrin thrombi in rats (12), in mice (13), and in rabbits (14) in the chronic phases. Furthermore, a human study found that the majority of macrophages expressed CD163 in aspirated fibrin thrombi from patients. Furthermore, the number of CD163 macrophages in fibrin thrombi was correlated with the time after onset. As a result, expression of CD163 macrophages may be a marker of fibrin thrombus age (11). CD163 has been proposed as a marker for assessing M2 macrophages distribution (15). Interleukin (IL)-6, IL-10, and glucocorticoids also increase the expression of CD163 in monocytes/macrophages. CD163-expressing macrophages may help to suppress an inflammatory response (16). That is, CD163-positive M2 macrophages should increase when acute inflammatory responses subside in the subacute and chronic inflammatory phases.

This case report has a limitation. As coronary angiography was not performed, the clinical diagnosis of ST-elevation myocardial infarction was presumptive.

In conclusion, the postmortem pathological evaluation successfully unveiled the clear pathophysiology of CCI, where intra-atrial thrombi due to AF induced concurrent cerebral-coronary artery embolism, although clear diagnosis in AIS and ST segment elevation in ECG was not completed. Further pathological examinations are required to establish clear pathomechanisms and preventive strategies of CCI.

## Data Availability

The raw data supporting the conclusions of this article will be made available by the authors, without undue reservation.
